# Duration of maternally derived antibodies of porcine parvovirus in growing pigs and presence of antibodies in gilts and sows vaccinated with three different parvovirus vaccines

**DOI:** 10.1186/s40813-024-00361-1

**Published:** 2024-04-09

**Authors:** René Renzhammer, Uwe Truyen, Birgit Buchebner, Gertrude Baumgartner, Rea Maja Kobialka, Ahmed Abd El Wahed, Michaela Koch, Andrea Ladinig, Christine Unterweger

**Affiliations:** 1 Department for Farm Animals and Veterinary Public Health, University Clinic for Swine, University of Veterinary Medicine, Vienna, Austria; 2 Institute for Animal Hygiene and Veterinary Public Health, University of Leipzig, Saxony, Germany

**Keywords:** Porcine parvovirus, ELISA, Haemagglutination inhibition test, Sows, Seroconversion, Maternally derived antibodies

## Abstract

While gilts and sows are regularly vaccinated against the porcine parvovirus (PPV), little is known on the presence of antibodies in vaccinated sows nor the decline of maternally derived antibodies (MDA) in their offspring. On twelve farms serum samples were taken from 180 gilts and sows vaccinated at least twice with one of three different commercial PPV vaccines. On nine farms, additional 270 serum samples were collected from growing pigs of three different age categories. All 450 samples were examined for PPV antibodies (Abs) by ELISA and haemagglutination inhibition (HI) assay. In total, 65% of all gilts vaccinated twice with either vaccine 1 or vaccine 3 were seronegative by HI assay. In each farm, there were at least three animals with high Ab titres (≥ 1:1280) indicating the presence of PPV in all twelve study farms. However, PPV DNA could not be detected in collected faecal samples. While low to moderately high Ab titres (1:10–1:640) were measured in 98% of twelve-weeks-old pigs, ELISA was only positive in 30% of the same pigs. Though, the statement on the duration of MDA may depend on the applied test, we could confirm an exponential decay of MDA. In addition, we could demonstrate that applied serological tools are insufficient for the confirmation of successful vaccination.

**Supplementary Information:**

The online version contains supplementary material available at 10.1186/s40813-024-00361-1.

## Background

The porcine parvovirus (PPV) 1 is considered as one of the most important infectious causes of reproductive failure in gestating sows [1, 2]. PPV is a small non-enveloped single-stranded DNA virus having a capsid which comprises of larger viral protein (VP) 1 molecules and smaller VP2 molecules [3, 4]. In general, PPV is ubiquitous in swine stocks worldwide [5]. While pigs of all ages can be infected with PPV, clinical signs predominantly occur in naïve gilts or sows being infected during gestation [6–8]. Depending on the stage of gestation, transplacental transmission of PPV1 can lead to a delayed return to oestrus, a decreased litter size and litters with increased numbers of mummified, autolyzed, and stillborn foetuses at the estimated date of birth or later, which is summarized by the term SMEDI (still birth, mummification, embryonic death, infertility) [3, 6, 7]. While PCR is carried out most frequently for direct detection of PPV-DNA in foetal tissues, serological tools are often applied to determine the antibody (Ab) status of gilts, sows and boars. Several ELISA and haemagglutination inhibition (HI) assay protocols have been established for the detection of PPV Abs [9–12]. While ELISAs are applied more frequently in routine diagnostic procedures, they do not permit a differentiation between vaccine derived Abs and Abs induced by infection with PPV [13]. On the other hand, HI assay results permit a certain interpretation, since PPV titres exceeding 1:1000 indicate previous infection with field virus, while titres ranging from 1:20 to 1:500 are presumably induced by vaccination [5, 14]. In most cases, Abs can be detected by HI assays approximately one week post infection and remain detectable for at least four years [15].

However, little data is available on seroconversion rates after vaccination, limiting the interpretation of PPV Ab results in vaccinated animals. In general, vaccines are applied frequently to protect gestating sows against disease caused by infections with PPV [5]. Currently, solely inactivated vaccines being distributed by five different companies are licensed in the European Union. While Parvoruvac® (Ceva Santé Animale, Libourne, France) is an inactivated whole virus vaccine based on the virulent Kresse-like strain K22, Eryseng®Parvo (Laboratorios Hipra, Amer, Spain), Suvaxyn®Parvo (Zoetis, Girona, Spain) and Porcilis®Ery + Parvo (Intervet International, Boxmeer, Netherlands) are inactivated whole virus vaccines based on avirulent NADL-2 or NADL-2 like strains [16–18]. In 2020, a novel subunit vaccine based on VP2 of PPV strain 27a (ReproCyc®ParvoFLEX, Boehringer Ingelheim Vetmedica GmbH, Ingelheim/Rhein, Germany) was launched [19]. In most cases, gilts are vaccinated twice between six and eight months of age and are consecutively revaccinated with a four to six months interval or once in every reproduction cycle [5, 16, 20]. In order to improve the interpretation of PPV Abs measured by ELISA and HI assay depending on the applied vaccine, we aimed to investigate Ab levels in gilts (after basic immunization) and sows of different parities in farms applying different PPV vaccines.

In addition, faecal samples were collected from the pen floors housing four-months-old fattening pigs in each farm to evaluate faecal shedding and consequently potential virus circulation in the farms. Since there is evidence that most gilts get infected with PPV prior to basic vaccination, interference of maternally derived antibodies (MDA) with antibodies due to active immunization cannot be excluded [21]. Therefore, knowledge on the actual time point of exposure to PPV and the duration of MDA in replacement gilts is pivotal for establishment of appropriate vaccination regimes. Consequently, we also aimed to evaluate the duration of MDA in those farms. In general, studies on the duration of MDA are inconsistent [15, 21, 22]. While it was originally demonstrated that PPV induced MDA last approximately until the fifth to sixth month of life, Gava et al. reported that MDA were no longer detectable by ELISA in serum samples of twelve-weeks-old pigs [15, 22]. Therefore, we hypothesized that MDA might be diminished prior to the sixth month of age. Thus, new vaccination time points in gilts can be reconsidered, since the late time point of basic immunization is based on the assumption of an interference between persisting MDA with vaccination [23].

## Results

### Reproductive data on study farms in the year prior to sampling

Within the year prior to sampling, the percentage of mummified foetuses, stillborn piglets and the return to oestrus rate varied among all twelve study farms (Table 1). While the percentage of mummified foetuses accounted for 4.2 in farms 9 and 12, the lowest percentage of mummified foetuses (0.5%) was observed in farm 6. Increased return to oestrus rates (> 10%) were observed in six farms.

**Table 1:** Reproduction data from all twelve study farms in the year prior to sample collection

Farm	Farm size N (sows)	Vaccine	Mummified foetuses (%)	Stillborn piglets (%)	Return to oestrus (%)
1	140	Vaccine 1 (Porcilis®EryParvo)	1.3	11.1	18.2
2	150		2.4	9.4	10.5
3	60		2.5	8.6	12.4
4	80		3.6	6.4	9.2
5	130	Vaccine 2 (Parvoruvac®)	2.1	9.5	11.5
6	166		0.5	10.3	2.5
7	150		1.6	3.5	9.0
8	75		0.3	4.8	9.6
9	65	Vaccine 3 (Eryseng®Parvo)	4.2	7.2	13.2
10	2.300		0.7	8.5	7.0
11	165		1.5	5.6	5.1
12	200		4.2	8.0	15.2

### Direct detection of PPV by PCR and virus isolation

PPV was neither detected by PCR nor virus isolation in investigated faecal samples collected in all twelve study farms.

### ELISA and HI test results from vaccinated sows and gilts

**Fig. 1:**
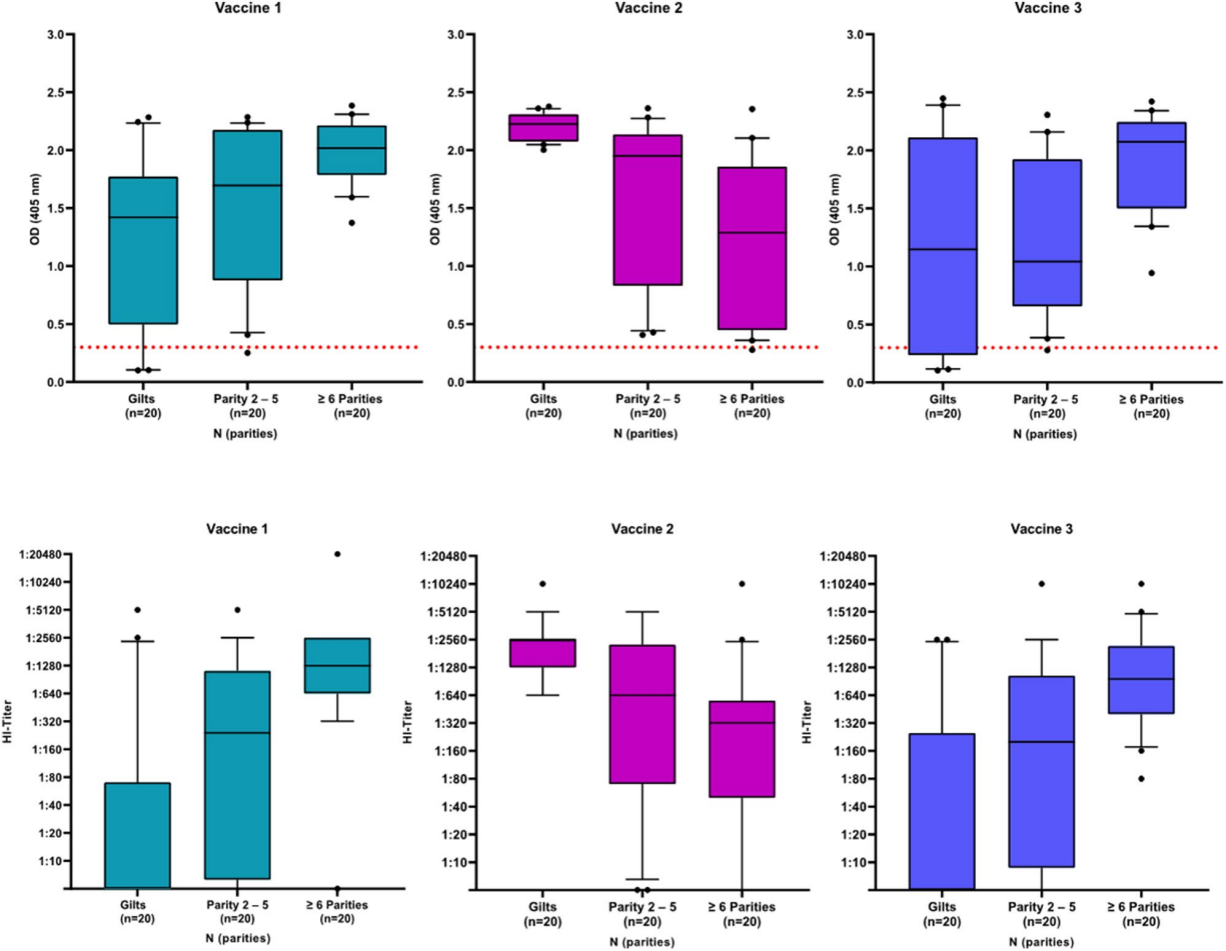
OD values and HI titres of serum samples from all 180 gilts and sows depending on parity and applied vaccine. OD: optical density; red dotted line: OD-value of 0.3 representing the threshold of the applied ELISA

Independent of the applied serological method, PPV Abs could be detected on a farm level, but not on an individual level. In total, samples from twelve gilts (20%) and three sows (2.5%) were negative by ELISA. Four of twelve seronegative gilts were vaccinated with vaccine 1, while the remaining eight seronegative gilts were vaccinated with vaccine 3. The three seronegative sows were vaccinated with vaccine 1, 2, and 3, respectively. No Abs were detected by HI assay in serum samples from another 27 gilts and sows, which were tested positively by ELISA. High HI assay Ab titres (≥ 1:1280) indicating previous infection with PPV were measured in 67 serum samples (37%) and in at least one sample from every farm. Ab levels of ≥ 6th parity sows vaccinated with vaccines 1 or 3 were higher compared to Ab levels of gilts vaccinated with the same vaccines (Fig. 1). On the contrary, gilts vaccinated twice with vaccine 2 had higher OD values and higher HI titres compared to sows regularly vaccinated with vaccine 2.

While ELISA results did not differ significantly among the different vaccines, Ab titres measured by HI assay were significantly higher in animals vaccinated with vaccine 2 compared to animals vaccinated with vaccine 1 *(p < 0,001)*, or vaccine 3 *(p < 0,001)*. The height of Ab titres by HI assay also differed significantly among the different age groups, as samples from ≥ 6th parity sows were significantly more likely to have high Ab titres compared to samples from gilts *(p < 0,001)*, or from sows in parities 2–5 *(p < 0,001)*. However, gilts vaccinated twice with vaccine 2 had significantly higher Ab titres compared to gilts vaccinated twice with vaccine 1 *(p < 0,001)*, or vaccine 3 *(p < 0,001)*. While Ab titres did not differ significantly among sows in parties 2–5 vaccinated with different vaccines, high parity sows vaccinated with vaccine 3 had significantly higher Ab titres compared to high parity sows vaccinated with vaccine 2 *(p = 0,022)*.

### Samples from sows and gilts vaccinated with vaccine 1

Abs were detected by ELISA in serum samples from 14/19 animals tested seronegative by HI assay. Since no Abs were detected by HI assay in serum samples from 13/20 gilts (65%), basic vaccination with vaccine 1 does not necessarily lead to formation of Abs detectable by HI. However, there is evidence that boosting sows with vaccine 1 leads to detectable Abs, as 15/40 sows had low to moderately high titres (1:10–1:320), whereas 6/40 sows were seronegative by HI assay. Altogether, 65% of ≥ 6th parity sows (13/20) had high HI-titres (≥ 1:1280) indicating a previous infection with PPV (Fig. 1).

### Samples from sows and gilts vaccinated with vaccine 2

Abs were detected by ELISA in all but one of the 60 serum samples. All gilts and 35/40 sows were seropositive by HI test. However, since Ab titres from 16/20 gilts accounted for ≥ 1:1280, there is evidence that most sampled gilts had been infected with PPV prior to sampling. Thus, no statement on seroconversion rate after basic vaccination with vaccine 2 can be done. Altogether, high Ab titres were measured in 27 serum samples (45%). In comparison to farms vaccinating with vaccine 1 or 3, ≥ 6th parity sows had lower Ab titres by HI test (Fig. 1).

### Samples from gilts and sows vaccinated with vaccine 3

All gilts from farm 9 were seronegative by both ELISA and HI test. In general, all ≥ 6th parity sows were positive by ELISA and HI assay. Since all gilts from farms 9, 10, and 11 were seronegative by HI test and all gilts from farm 12 had high HI titres indicating a previous infection with PPV, there is evidence that basic vaccination with vaccine 3 does not lead to formation of Abs, which are detectable by HI assay. High antibody titres were measured in samples from sows in all four farms indicating presence of PPV field virus (Supplementary Material Table S1).

### Antibodies in growing pigs

**Fig. 2:**
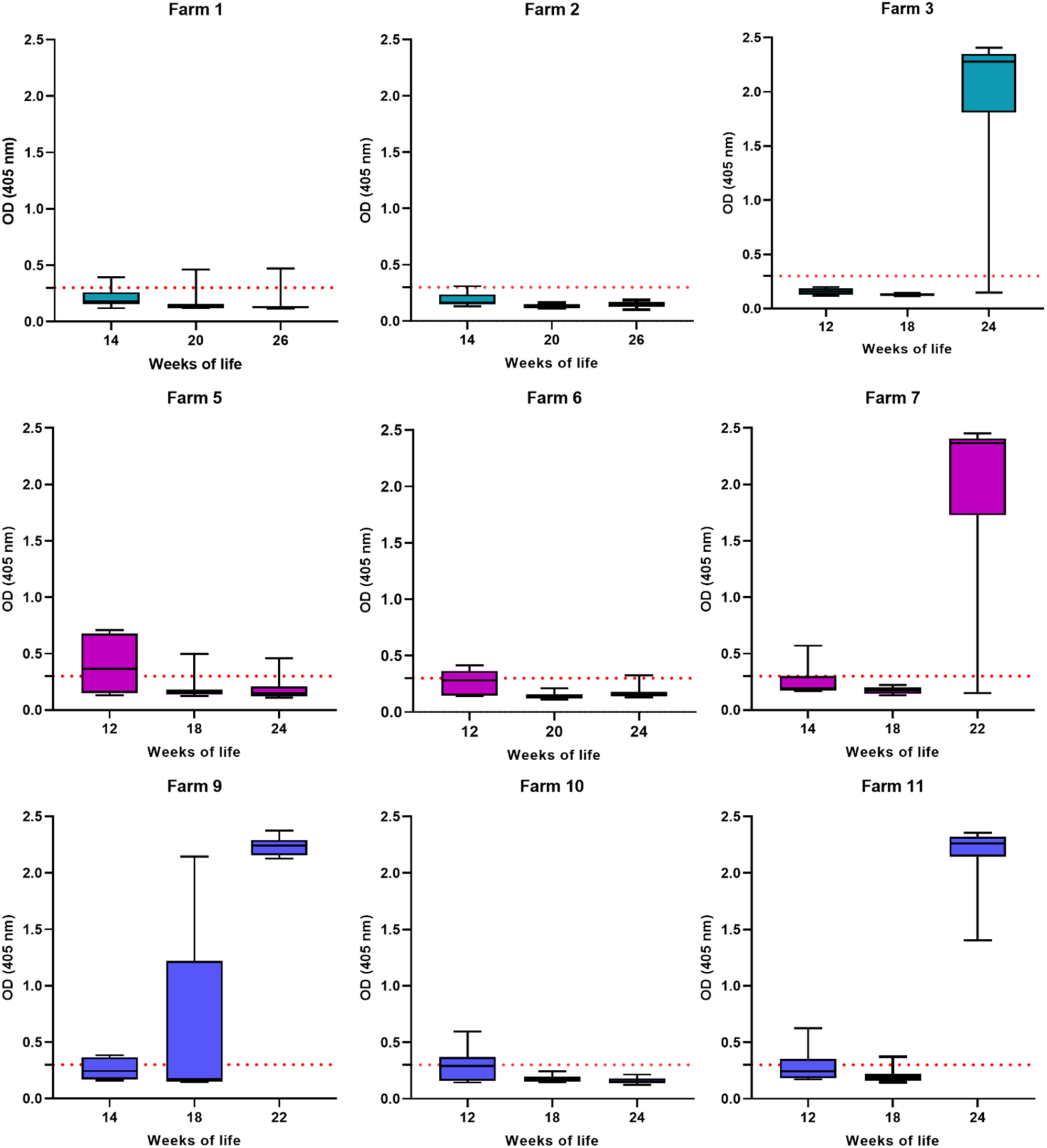
OD values of serum samples from all 270 growing pigs depending on the age and farm and vaccine applied to the mother sows. OD: optical density; red dotted line: OD-value of 0.3 representing the threshold of the applied ELISA

Altogether, 14% of all growing pigs were positive by ELISA. A similar decay in OD values from younger to older pigs was observed in serum samples taken on five farms (farms 1, 2, 5, 6 and 10) (Fig. 2). While 30% of sampled three-months-old pigs were positive by ELISA, only 8% were positive by ELISA by the age of 18 to 20 weeks. OD values of samples taken in farms 3, 7, and 11 similarly started low and decreased further until the age of 18 weeks. In contrast to the other farms, a sudden rise in OD values in the oldest group was observed in those three farms (Fig. 2). On farm 9, high OD values were already measured in three samples from 18-weeks-old pigs.

**Fig. 3:**
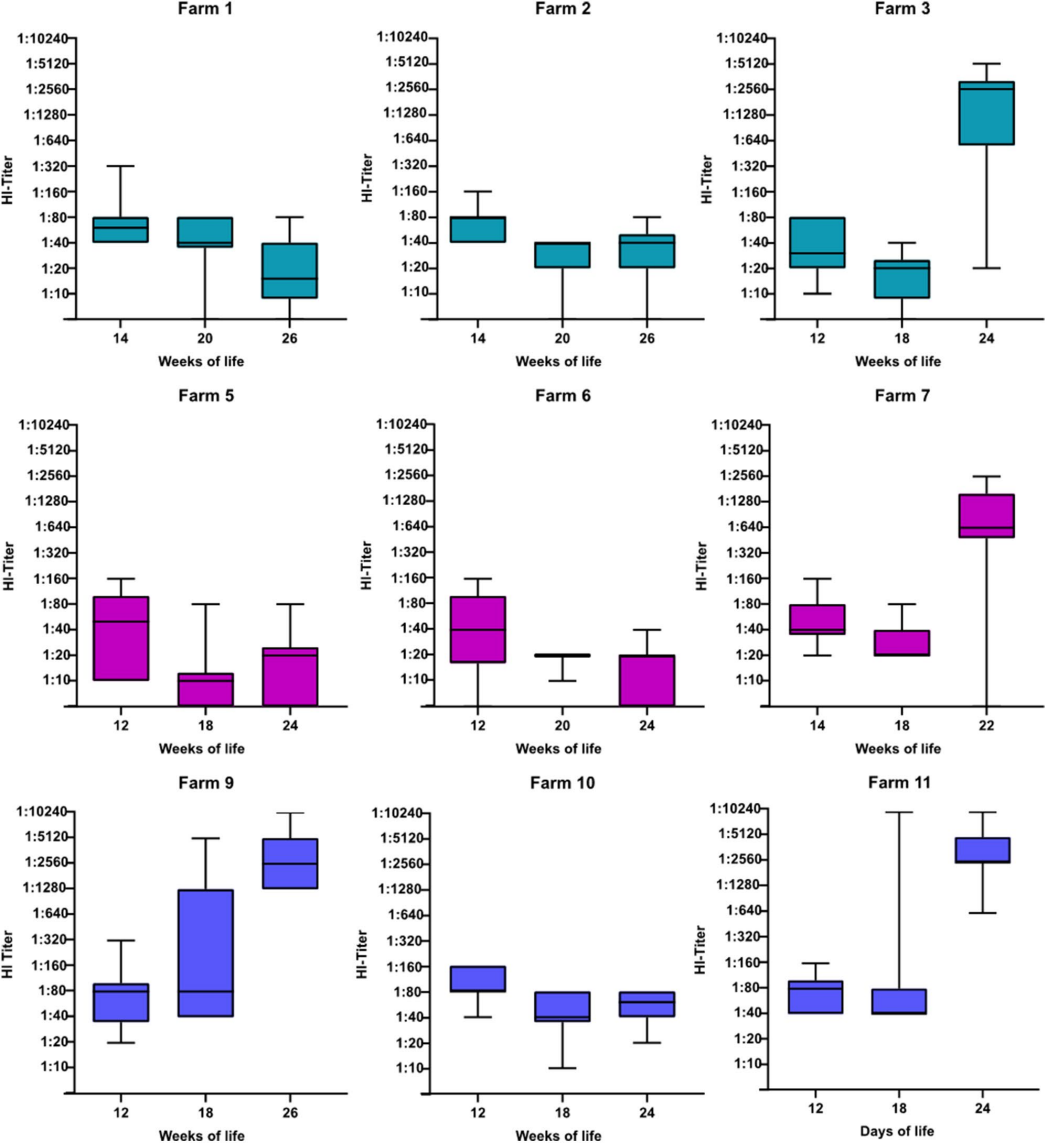
HI titres of serum samples from all 270 growing pigs depending on the age and farm

Altogether, PPV Abs were detected by HI test in 250 of 270 (93%) investigated samples from growing pigs. PPV Abs were detected by HI test in 98% of all samples from three-months-old pigs. Similar to ELISA results, a decline in Ab titres was observed from three to six-months-old pigs on farms 1, 2, 5, 6, and 10 (Fig. 3). However, in contrast to ELISA results, low to moderately high levels of PPV Abs (1:10–1:640) were still present in most samples of six-months-old pigs from those five farms (41/50). Besides three samples from 18-weeks-old fattening pigs from farm 9, high Ab titres (≥ 1:1280) were solely measured in samples from six-months-old pigs from farms 3, 7, 9, and 11.

## Discussion

In general, our data clearly emphasize the problems in the interpretation of serological test results which may be attributed to several reasons. First of all, serological tests are inappropriate for the confirmation of the involvement of PPV in reproductive problems, as high HI Ab titres or high OD values are not associated with reproductive disorders of sows associated with PPV infections [24]. However, reproductive disorders varied among farms. For example, in farm 12 an increased number of mummified foetuses was observed in November prior to sampling. Nevertheless, investigated mummified foetuses from farm 12 were negative for PPV DNA. In addition, PPV as cause of increased return to oestrus rates in six farms could not be excluded, since there are no appropriate diagnostic measures besides post-mortem investigations of the rebreeding sows. While we also observed a high variation of stillborn piglets, stillbirths may be also attributed to a variety of non-infectious causes [25]. In addition, there is no evidence that absence of measurable Abs predisposes gestating sows for symptoms associated with PPV, since sufficient protection also depends on the presence of serum neutralizing Abs and cellular immunity, which were not tested in the current study [26]. Furthermore, the interpretation of serological results depends on the applied assay, as Abs were detected by INgezim® PPV ELISA in over two thirds of all gilts and sows tested seronegative by HI assay. Therefore, it is possible that this ELISA is more sensitive in measuring vaccine derived Abs compared to HI assay. However, results may vary depending on the applied ELISA. In addition, results of HI assays also depend on the used antigens. Since there is currently no knowledge on PPV strains circulating predominantly in Austrian swine stocks, it is possible that applied antigens for HI assay and field antigens differ.

In particular, vaccination of animals further aggravates the interpretation of serological tests. While we could demonstrate the absence of PPV Abs by HI in most gilts after receiving the basic vaccination with vaccine 1 or 3, a statement on the seroconversion rate in gilts vaccinated twice with vaccine 2 cannot be provided due to the presence of high Ab titres indicating a previous infection with PPV [5].. Similar to our results, low seroconversion rates by ELISA in gilts vaccinated twice with vaccine 1 or vaccine 3 were already reported before [21]. Van den Born also reported that PPV Abs were detected by HI assay in only one third of all gilts vaccinated with vaccine 1 [18]. However, it was previously demonstrated that vaccination of gilts with vaccine 2 solely induced low Ab levels measured by INgezim® PPV as well [17]. Although the applied HI assay, vaccine 1 and vaccine 3 are all based on strains closely related to NADL-2, most sampled gilts vaccinated twice with vaccine 1 or 3 had no or low Ab titres by HI test. However, most older sows vaccinated with vaccine 1 or 3 were seropositive by HI test. Therefore, we assume that regular revaccination leads to high levels of vaccine derived Abs. This goes along with observations of Sánchez-Matamoros et al. reporting a significant boost in measurable Abs after the third vaccination of sows with vaccine 3 [16]. Thus, vaccination intervals could potentially be extended in older sows, if technically feasible. However, this hypothesis should be proven, as we cannot exclude that high Ab titres were the result of previous infections with PPV field virus.

Furthermore, interpretation of serological results in gilts under field conditions may be even more difficult since in Austria they usually receive their first vaccination on nucleus herds and their second vaccination in the piglet producing farm. Therefore, application of two different vaccines is possible. However, there are currently no studies on the seroconversion rate of gilts vaccinated twice with two different PPV vaccines. Thus, application of serological PPV assays is not applicable for monitoring or routine diagnostic procedures in vaccinated herds.

Since titres exceeding 1:1280 were measured by HI test in samples deriving from all twelve farms, it is likely that PPV had been circulating in all study farms. In general, our results on high Ab titres go in line with results from a Finnish prevalence study reporting the detection of high Ab titres in 17/21 investigated farms and 44% of sampled sows [24]. Nevertheless, we could not detect PPV directly by PCR or virus isolation in collected pooled faecal samples. This could have several reasons. Firstly, we might have missed rectal shedding in the sampled animals, since PPV is usually shed via faeces exclusively within the first three weeks after infection [20]. In addition, as high Ab titres were measured in serum samples from six-months-old pigs, but faecal samples were collected from four-months-old pigs, it is likely that we collected faeces from the wrong age group. Since we did not perform sample size calculation to estimate the number of faecal samples to be collected for the detection of PPV, the overall sample size might have been too small. Furthermore, circulation of PPV may predominantly occur in farm compartments with mainly naïve animals [6]. Nevertheless, since high Ab titres were measured by HI in samples from all twelve farms, vaccination of gilts is still indicated to prevent reproductive problems caused by PPV infections. Hence, despite the evidence of PPV infection of gilts prior to sampling in several study farms gilts, there were still farms with seronegative gilts emphasizing that solely relying on circulation and active immunization of gilts due to field virus contact prior to insemination is too risky.

While we could demonstrate that low levels of MDA may at least last until six months of age, we observed big differences in the decay of MDA and rise of Abs probably due to infection among different herds and individuals. In general, recent literature on the duration of MDA is scarce and mainly opposes common knowledge which is predominantly based on literature from the 1970s [21, 22]. In accordance with a Brazilian study, most sampled growing pigs were seronegative by ELISA [22]. However, Abs were detected by HI test in 93% of all samples from growing pigs, indicating a longer persistence of MDA. Thus, statement on the duration of MDA depends on the applied serological test and selected thresholds. For example, in previous investigations reporting an earlier decline of MDA, a threshold of 1:320 for HI was selected, or solely ELISA was applied for measurement of MDA [21, 22]. In addition, we could also observe an asymptotic decline of MDA in growing pigs from three to six months of age. However, Ab titres may increase after infection with PPV field virus, which was reported to happen after the 22nd week of life [21]. This goes along with our results, as in three farms (farms 3, 7, and 11) Ab titres exceeding 1:640 were only measured in serum samples from six-months-old pigs. Our data on the decay of MDA in nine different farms emphasize that decay of MDA and time point of infection vary among the farms, which may be attributed to several reasons. Firstly, an early PPV infection of growing pigs may be the result of high PPV viral loads due to poor management and a high pig flow. For instance, in all four farms with high Ab titres in six months-old pigs [3, 7, 9, 11] there was no consequent separation of production units compared to farms 2, 4, 5, 6 and 10. In addition, decay of MDA also depends on the height of Abs on the first day of life. Therefore, the colostrum intake of piglets and the overall amount of Abs in the sows after parturition also have an impact on the duration of MDA. The high variation in Ab titres detected by HI test of sows in the same farms, which might be related to the genetic background of outbred pigs, may partially explain the high variation of Ab titres in their offspring. Therefore, results on MDA may also depend on the mother sows of sampled piglets.

Despite the limitation in sample size, we assume that choice of PPV vaccine used on the sows has little impact on the height of MDA in their offspring, as the height of Ab titres of three-months-old fattening pigs did not differ among farms.

It was described that an infection with PPV predominantly occurs three to five weeks after the decay of MDA [22]. However, we could not confirm that solely the decay of MDA predisposes pigs for an infection with PPV, as all 18-weeks-old fattening pigs in farm 7 still had measurable Abs by HI, while pigs which were four weeks older had high titres indicating a PPV-infection. Consequently, we assume that MDA may not block immune response after infection with PPV. Therefore, our data provide evidence that replacement gilts can be vaccinated against PPV prior to six months of life despite the presence of low levels of MDA. While vaccinating gilts prior to their 180th day of life would be convenient, there are currently no vaccines licensed for vaccinating pigs before their 180th day of life and novel registrations of licensed PPV vaccines would require expensive experimental trials in sows.

One of the major limitations of the study was that no longitudinal sampling was performed. In general, longitudinal sampling could have helped to receive better information on the actual duration of MDA in growing pigs compared to cross-sectional sampling. In addition, taking at least paired serum samples of gilts and sows could have helped to distinguish better between Abs due to previous infection and vaccine derived Abs by HI. Nevertheless, in the field animals are frequently sampled once for monitoring purposes and to confirm the potential involvement of PPV in fertility problems [5]. Therefore, we aimed to evaluate, if the interpretation of PPV Abs is feasible in animals sampled only once. Another weakness is the limited comparability of serological results due to the high variation in weeks between vaccination of pigs and sampling among study farms. In addition, the actual age of sampled gilts and sows was not provided. However, since gilts were first inseminated by the age of 7.5–9 months in all farms and rebreeding sows were not sampled, the number of parities is very likely to represent the actual age distribution of sampled animals.

Despite the fact that our data emphasize the problems of serological assays for PPV Abs, they also allow insights into the dynamics of PPV Abs in different age groups of piglet-producing farms.

## Conclusions

There is evidence that low levels of MDA may persist for at least up to six months of life, but not necessarily do that. There is currently no evidence of vaccine-derived Abs in gilts vaccinated twice with vaccine 1 or vaccine 3. While high Ab titres were measured by HI in samples in most farms, PPV could not be detected directly in faecal samples collected from four-months-old pigs. Altogether, serological results are difficult to interpret and should be avoided if direct detection of PPV-DNA can be applied instead.

## Methods

### Sample size calculation

We aimed to evaluate the prevalence of gilts and sows which were negative for PPV Abs by ELISA despite vaccination for each vaccine (*n* = 3) individually. As two vaccines (Suvaxyn®Parvo and ReproCyc®ParvoFLEX) were not licensed in Austria at the time point of sampling, we had to exclude them from further investigation. Sample size calculation for sampled sows was based on a confidence level of 95%, an accuracy of 20%, an expected prevalence lower than 20% and a population size of 2300. The expected prevalence of seronegative sows was based on the evaluation of ELISA results from serum samples sent to the University Clinic for Swine for PPV Ab detection during routine diagnostic procedures from 2018 to 2020. The population size was based on the largest participating piglet-producing farm with 2300 sows. Therefore, the sample size accounted for 15 animals.

Sample size calculation for growing pigs was based on a confidence level of 95%, an accuracy of 20%, an expected prevalence lower than 10% and a population size of 1500 resulting in a minimum sample size of nine animals. However, we decided to increase the number of sampled animals to ten. The expected prevalence was based on the results of Gava et al. and Anderson et al. reporting a prevalence of 1.5% seropositive growing pigs by the age of three months (29, 30). The population size was based on the largest farm having in average 1500 twelve weeks-old pigs per batch. Sample size calculation was not performed to estimate the number of faecal samples to be collected for the detection of PPV shed by four months old pigs.

### Sampling

In June 2021, serum samples from 15 animals each were collected in twelve Austrian piglet producing farms (Table 2). In each of four farms, the same inactivated whole virus PPV vaccine was applied by the respective herd veterinarian to vaccinate gilts and sows for over five years prior to sampling. Vaccine 1 (Porcilis® EryParvo, Intervet, Boxmeer, Netherlands) was applied in farms 1–4, vaccine 2 (Parvoruvac®, Ceva Santé Animale, Libourne, France) was applied in farms 5–8, and vaccine 3 (Eryseng®Parvo, Laboratorios Hipra, Amer, Spain) was applied in farms 9–12.

In every farm, serum samples were taken from (a) five gilts, (b) five sows from parities 2–5, and (c) five sows from parities 6–15 (Table 2). At the time point of sampling, all gilts were vaccinated twice with the same vaccine at least 14 days prior to sampling. All sows were revaccinated at least twice a year, but vaccination regime varied amongst farms (Table 2). While rebreeding sows were not sampled, samples from sows were always taken within the first four weeks after insemination from sows housed in the service centre. Besides serum samples, ten faecal samples were collected and pooled from the floor of one pen with four-months-old growing pigs in every farm for the detection of PPV DNA shed via faeces.

**Table 2:** Study farms and vaccination regimes against the Porcine Parvovirus

Farm	Farm size N (sows)	Vaccine	Number of sampled gilts and sows	Number of sampled growing pigs	Number of weeks between last vaccination and sampling	Age of gilts at the time of 1st and 2nd PPV vaccination (weeks of life)	Rearing own gilts
1	140	Vaccine 1 (Porcilis®EryParvo)	15	30	17	29th + 32nd	Yes
2	150		15	30	24	26th + 30th	No
3	60		15	30	18	26th + 30th	Yes
4	80		15	-	7	26th + 30th	No
5	130	Vaccine 2 (Parvoruvac®)	15	30	*	26th + 29th	No
6	166		15	30	*	26th + 29th	Yes
7	150		15	30	*	32nd + 35th	Yes
8	75		15	-	*	32nd + 35th	No
9	65	Vaccine 3 (Eryseng®Parvo)	15	30	9	26th + 29th	Yes
10	2.300		15	30	14	26th + 29th	Yes
11	165		15	30	5	26th + 29th	Yes
12	200		15	-	14	26th + 30th	No

*sows are vaccinated during the lactation period, PPV: porcine parvovirus

Additional 30 serum samples were collected from fattening pigs (farms 1, 2, 3, 5, 6, 9, 11) or replacement gilts (farms 7 & 10) in nine of twelve farms. Serum samples were not taken from the remaining three farms, since no growing pigs were kept on site. In each farm, serum samples were collected from ten pigs between ten and twelve weeks of age, ten pigs between 18 and 20 weeks of age, and ten pigs between 22 and 26 weeks of age.

## ELISA

Abs specific for the VP2 of PPV1 were measured in all 450 serum samples using a commercial, indirect enzymatic Immunoassay (INgezim® PPV 0.11.PPV.K1, Ingenasa, Madrid, Spain) according to the protocol provided by the manufacturer. Based on the manufacturer’s instruction, samples with an OD value exceeding 0.3 were considered as positive, whereas samples with OD values ≤ 0.3 were considered as negative.

### Haemagglutination inhibition assay

Haemagglutination inhibition assay was performed as descried previously using 0.5% human erythrocytes (O, rhesus-negative, ethical approval: ID: 068/23-ek, Ethics Committee at the Medical Faculty of the Leipzig University, Germany) [2]. Embryonic porcine kidney epithelial cells (SPEV cells) were provided by the Bio Bank of the Friedrich-Loeffler-Institute (Insel Riems, Germany) and used for virus isolation. The latter was performed using PPV NADL-2 strain. Based on previous findings, Ab titers < 1:10 indicated that the animal has not seroconverted while titers ≥ 1:1280 were considered as high titers indicating previous infection with PPV [13].

### Molecular test

DNA extraction was conducted via DNeasy Blood & Tissue Kits (Qiagen, Hilden, Germany) as described by manufacturer. Real-time PCR targeting Ungulate protoparvovirus 1 was performed according to Streck et al. [27]. The master mix of the 2x qPCR BIO Probe Mix No-ROX (PCR BIOSYSTEM, London, England) was used per manufacture guidance on Mx3000P platform (Stratagene, La Jolla, USA) using the following thermal profile: activation at 95 °C for 3 min, 45 cycles of 95 °C for 10 s and 60 °C for 25 s.

### Virus isolation from fecal samples

Virus isolation was performed by adding 100 mg of fecal samples to five times volume of Dulbecco’s Modified Eagle Medium (DMEM, *Biowest, Nuaillé, France*) supplemented with 200 U streptomycin (*Gibco, Waltham, Massachusetts, United States)* [26, 28]. The supernatant was passed through 20 μm filter after three cycles of freezing and thawing and centrifugation at 2000 g with temperature kept at 4 °C for 20 min. Hemagglutinin activity using 0.5% human erythrocytes was screened before inoculation onto SPEV cells. Cultures were screened for up to 6 days for a CPE. For immunofluorescence, cells were fixed with acetone/methanol (1:1) on day five post infection and visualized using fluorescein isothiocyanate-conjugated anti PPV antibody (Veterinary Medical Research & Development, Pullman, United States).

### Statistical analysis

All statistical analyses were performed in R version 4.3.2 [29]. Two generalized linear models were calculated, one with Ab titres by HI assay and one with ELISA results (positive/negative) as the output variable. The number of litters, the vaccine and the interaction of both were defined as explanatory variables while the farm was set as a random effect applying the function *glmer* in package *lme4*. After model calculation, post hoc tests were performed using the function *glht* in package *multcomp*.

### Electronic supplementary material

Below is the link to the electronic supplementary material.

Supplementary Material 1

## Data Availability

All collected data are available as supplementary material.

## Abbreviations

PPV: porcine parvovirus
VP: viral protein
Ab: antibody
HI: haemagglutination inhibition
MDA: maternally derived antibodies
